# What school nurses receive for themselves that influences their remaining in practice: A qualitative study

**DOI:** 10.1186/s12912-023-01229-5

**Published:** 2023-03-22

**Authors:** Linda Horne Mæland, Bjørg Frøysland Oftedal, Margareth Kristoffersen

**Affiliations:** Faculty of Health Science, University of Stavanger, PO Box 8600, 4036 Forus, Stavanger, Norway

**Keywords:** Work-life, Good life, Nursing practice, Remaining in practice, School nurse, Phenomenological hermeneutic method, Hermeneutic approach

## Abstract

**Background:**

Previous research indicates a link between what nurses receive for themselves and their remaining in practice. In Norway, school nurses tend to remain in practice, but what it is they receive for themselves has been scarcely studied. The aim of this study, therefore, was to describe and interpret what it is school nurses receive for themselves that influences their remaining in practice.

**Method:**

The study has a qualitative design with a hermeneutic approach. Data were collected through individual interviews on two separate occasions with 15 Norwegian school nurses. The data were analysed using a phenomenological hermeneutic method.

**Results:**

Two themes demonstrate what it is the school nurses receive for themselves: (1) ‘Gaining interesting workdays for yourself’ and (2) ‘Attaining pleasure for yourself’. Each theme has two sub-themes. The first theme involved the school nurses ‘having an attractive scope of practice’ and ‘having varied tasks’. The second theme involved ‘being trusted’ and ‘being given a response’. The study themes can be comprehensively understood as an expression of what the school nurses identify as the main locus of the good work-life. The school nurses’ remaining seems to revolve around what it is they receive on their own behalf: an affirmation for their ordinary life and what they do as a nurse.

**Conclusion:**

This study highlights that what school nurses receive on their own behalf may influence their remaining in practice. It adds to previous research with a more specific understanding of nurses remaining in practice by stating that in identifying the main locus of the good work-life, the school nurses received affirmation for their ordinary life and what they do as a nurse. Thus, it is important that nurses identify the main locus of a good work-life for themselves, as receiving affirmation for what they do in their ordinary workdays may influence their remaining in practice.

**Registration of clinical trial and registration identification number:**

The study was approved by the Norwegian Centre for Research Data (project 59195). National Research Ethics Committee approval was not required, as the study only involved health professionals and did not ask for sensitive information.

## Background

The international nursing shortage is estimated to be 7.6 million nurses by 2030 [1], which is an alarming figure, and partly due to the recent years of the pandemic adding even more pressure on the nursing profession [2]. Around the world there is a rising demand for nurses, as one in six nurses is expected to retire by 2030 [1, 3] and nurses are leaving the profession [4–6]. In Norway, one in five nurses leave their profession during the first ten years of practice [7]. However, Norwegian school nurses are a group who tend to remain [8]. It is therefore highly relevant to look more closely at what it is they receive for themselves that may influence their remaining in practice.

Research indicates that nurses receive something in exchange for what they give to patients. Nurses are more willing to unconditionally engage in and take responsibility for nursing care, if they receive a “sense of reciprocity” in everyday nursing practice (9, p. 7). Providing what is good for the patients seems to be good for the nurses themselves [10]. This was also reported by Florence Nightingale, the British nurse and foundational philosopher of modern nursing, who in 1860 stated that a nurse looks after her patients “for her own satisfaction” (11, p. 198). Nurses receive recognition from patients, their families or colleagues for the care they provide [10]. By receiving recognition, nurses acknowledge their success as a nurse and become proud of themselves [12, 13]. This gives them a zest that is possible to feel on their own behalf [10, 14, 15]. Nurses can also experience a sense of well-being, described as ‘muchness’, in a nursing practice with opportunities to creatively realize nursing care in accordance with professional nursing values [16]. Such opportunities have an impact on how nurses thrive and perceive their work-life as meaningful for themselves [15, 16]. What is meaningful here relates to professional development, which implies they keep up with professional knowledge and skills [14, 15, 17]. Thus, what nurses receive for themselves can give an indication of what influences their remaining in practice [18–20].

In Norway, school nurses are Registered Nurses with post graduate education in Public Health Nursing. School nurses are employed by the municipalities, which are legally obliged to offer a school health service for all public and private schools, from elementary to high school. Like their counterparts in other parts of the world, the school nurses’ responsibility involves health promotive and disease preventative interventions for school children’s health and well-being. School nurses are regularly in attendance at schools and are engaged in activities such as health education, immunization, screening, and individual counselling [21, 22]. They provide support for school children with a diversity of problems that range from obesity, self-harm, domestic violence, and sexual health and identity. School nurses offer nursing care for school children with diagnosed conditions and complicated requirements, and also collaborate with families, caregivers and teachers to ensure school children’s needs are addressed [23, 24]. School nursing is described as a challenging part of nursing practice, and school nurses report emotional exhaustion due to a high workload and ethical dilemmas [25, 26]. Often, they deal with issues like child abuse, thoughts about suicide or family problems, and risk damaging a trusting relationship with school children when they break their confidentiality by informing parents or the child welfare service about a situation [27, 28]. In addition, school nurses feel expended over a broad scope of tasks and perceive a lack of time and ability to meet the obligations or complex health needs of school children [29, 30]. However, researchers report that having an impact on school children’s life-long health habits is a positive aspect of the school nurses’ work-life [31, 32]. Indeed, making a difference to the children’s lives is something school nurses often perceive as an accomplishment, and this may contribute to their sense of satisfaction and remaining in practice [33]. Nevertheless, there has scarcely been any research as to what school nurses receive for themselves that influences their remaining in practice.

### Aim

The aim of this study was to describe and interpret what it is school nurses receive for themselves that influences their remaining in practice.

## Methods

### Design

This article is part of a larger study about self-realization and its influences on school nurses’ remaining in practice [34, 35]. This current study has a qualitative design [36] with a hermeneutic approach [37], which involves describing what the text says and interpreting what it means. This was done to clarify the underlying meaning of the text. Two individual in-depth interviews [38] with each participant were conducted and a phenomenological hermeneutic method [39] was chosen. The method was considered appropriate for the analysis of what it is the school nurses receive for themselves.

### Setting and recruitment

This study recruited participants by purposive sampling [36] with the following inclusion criteria: registered nurse holding a post graduate qualification in Public Health Nursing, current position as a school nurse in full or almost full time employment (75–100%), and a minimum of three years’ work experience as a school nurse. The heads of the school health service in 39 municipalities in southern Norway were contacted and asked to invite school nurses to participate in the study. A total of 15 school nurses contacted the first author and gave their consent to participate. All the participants were female and represented 12 municipalities. Their ages ranged from 33 to 60 (median 48), with work experience as a school nurse ranging from 4 to 20 years. Of the 15 participants, 11 had one or more additional postgraduate level qualifications related to mental health, psychosocial work, paediatrics, midwifery, intensive care, leadership, family therapy, or counselling.

### Data collection

The individual in-depth interviews took place in undisturbed locations selected by the participants. An interview guide was used, and the participants were asked to talk freely about what it is they receive for themselves that influences their remaining in practice. To elicit more details from the participants, open-ended follow-up questions were posed: ‘I would like to hear more about what it is you receive for yourself’, ‘What does this mean for you?’, ‘What influence may this have on your remaining in practice?’, and ‘What more can you tell about what you receive for yourself that influences your remaining in practice?’ The same introductory open-ended question was asked in the second interview so that the participants could elaborate on or add to their first reflections. The average duration of the interviews was 70 min for the first interview and 40 min for the second. Data were collected from April-August 2018 by the first author and comprised a total of 30 interviews, which were audiotaped and consecutively transcribed verbatim.

### Ethical considerations

The heads of the school health services were provided with oral and written information about the study in order to invite school nurses to participate. Potential participants were given an information letter about the study detailing the estimated time for the two interviews, assurance of confidentiality and anonymity and the right to withdraw at any time. All the participants signed an informed consent before the first interview. The study was approved by the Norwegian Centre for Research Data (project 59195). Data have been stored and handled according to research ethical guidelines [40].

### Data analysis

The phenomenological hermeneutic method for analysis involved a dialectic movement between parts and the whole of the interview material in three phases: naïve reading, thematic analysis, and comprehensive understanding [39]. The first phase was naïve reading, which involved listening to the recorded interviews and repeatedly reading the transcribed data with an open mind to gain a preliminary understanding of what the participants receive for themselves that influenced their remaining in practice. The preliminary understanding was formulated as follows:

“What the school nurses received for themselves that influences their remaining in practice was workdays which they enjoyed”.

In the second phase, a thematic analysis was performed and involved de-contextualizing the data material and identifying meaning units. Guided by the preliminary understanding, analytical questions were posed: ‘What did the school nurses enjoy about their workdays?’ and ‘From whom and how did the school nurses receive what made them enjoy their workdays?’ To condense the text and facilitate the analysis further, reflective questions were posed to the text: ‘What does this involve?’, ‘What is this about?’, and ‘What does this mean?’ Meaning units were sorted by searching for similarities and differences in the text. The underlying meaning of the text was then reflected on and discussed among all authors, which resulted in validation of the preliminary understanding and formulation of themes.

The last phase of the analysis involved an overall interpretation of what the nurses received for themselves that influenced their remaining in practice. All authors reflected on the preliminary understanding and the formulated themes in regard to the study’s aim and background to reveal a comprehensive understanding.

### Rigour

Rigour was ensured by following the criteria for trustworthiness: credibility, confirmability, dependability and transferability [41]. To ensure credibility, the study facilitated two in-depth interviews with each participant to give them time to mature their reflections on their daily experience in regard to the study’s aim. Moreover, this facilitated the creation of a detailed account with nuances in the data material and contributed to prolonged engagement with the participants, opening up a trustful atmosphere for them to talk freely. Although all the interviews were performed by the first author, confirmability was ensured, as the transcripts of the first interview were discussed among the authors to consider proximity in the interview situation. No changes considered relevant were made to the interview guide, and sufficient questions were asked to achieve rich data. All authors took part in all analytical stages. The condensed text was discussed until consensus was reached on the analysis and the formulated themes. This strengthens the dependability of the study, together with a clear description of the research process. The results are presented with participants’ quotes to facilitate external judgement and to consider transferability of results to other nursing contexts.

## Results

The thematic analysis revealed two themes: (1) Gaining interesting workdays for yourself, and (2) Attaining pleasure for yourself.

### Gaining interesting workdays for yourself

The theme ‘Gaining interesting workdays for yourself’ consists of two sub-themes: ‘Having an attractive scope of practice’ and ‘Having varied tasks’.

#### Having an attractive scope of practice

Having an attractive scope of practice refers to a nursing practice the school nurses liked and was within their interest. School nursing was seen as a unique scope of practice where they gained interesting workdays for themselves. The nurses’ workdays involved meeting with school children, supporting them in achieving a healthy lifestyle and preventing more serious health problems. One nurse who previously worked in mental health care articulated her attraction to this scope of nursing practice:

When I worked in psychiatry, I thought that it might be possible to prevent some serious problems. Nursing practice in a high school is a unique place for early intervention, with a focus on health and well-being. The school children are young people who are on the threshold of life. Basically, they are healthy with some everyday problems, though some problems are more serious than others, and I like being able to provide them with hope for the future (School nurse 10).

This quote demonstrates why school nursing had an attractive scope of practice. During their workdays, the nurses provided the school children with hope for the future and helped them cope with the everyday or more difficult problems. Such a nursing practice was purposeful for the children’s life and upbringing and meaningful for the nurses themselves. One nurse clearly expressed that it was so meaningful to be able to give help and positively influence the school children’s health, that it was ‘painful’ to her if she was not able to do so:

It is really meaningful for me to plan and provide health preventive work for school children. I meet them, talk with them, provide them with information, and in this way I help them to maintain good health and make good choices for themselves. It feels good for me to help them to help themselves. It is painful to observe school children who make choices that are unfortunate or harmful to themselves. (School nurse 9)

It was good for the nurses to provide the school children with the nursing care they needed, which indicates a connection between the scope of practice and the nurses gaining interesting workdays for themselves. This may be a reason for their remaining in practice.

#### Having varied tasks

Having varied tasks contributed to the school nurses gaining interesting workdays for themselves and influenced their remaining in practice. A typical workday consisted of a wide range of varied tasks, ranging from individual consultations to teaching sessions and tutoring for teachers or parents. The nurses liked constantly being presented with new and unexpected tasks. Sometimes they were taken by surprise when school children turned up at their door, and they found it exciting to explore how to tackle specific problems they were told about in the situation. One nurse associated herself with MacGyver, the American action-adventurer and quick-witted TV-hero who explores and solves all types of situations. She said:

I like that I don’t know who’s coming to my door, that there’s always something new happening. I have to be like MacGyver, ‘what’s up?’ and ‘what to do here?’ Sometimes it may be stressful or chaotic, but it’s a positive challenge I find necessary to prevent becoming bored at work. (School nurse 7)

For this nurse, it was exciting to have varied tasks, which also represented challenges, as there was no ‘quick fix’ for how she could tackle the different problems the school children brought to her. Although varied tasks could involve stressful or chaotic workdays, they nevertheless made work interesting and prompted the nurses to use all their skills and qualities as a nurse. Such variety prevented them from becoming bored at work.

Moreover, having varied tasks involved the nurses collaborating in interdisciplinary teams with teachers, the municipality child welfare service, the police, and other health care professionals. This interdisciplinary collaboration was reported as inspiring and instructive, as the nurses could share experiences and knowledge across disciplines and were thus able to influence, correct and adjust their efforts to help the school children. One nurse said:

As I attend meetings with other professionals, I get to know their perspectives. It makes me feel privileged, and I learn to understand there are other solutions than what I first thought. It makes me see more nuances, different opportunities and solutions, but also more limitations. (School nurse 5)

The interdisciplinary collaboration was perceived as a privilege, as it gave a wider understanding and insight into a school child’s situation, which in turn kept the nurses interested in their work-life.

### Attaining pleasure for yourself

The theme ‘Attaining pleasure for yourself’ consists of two sub-themes: ‘Being trusted’ and ‘Being given a response’.

#### Being trusted

For the school nurses, being trusted was one source of attaining pleasure for themselves that influenced their remaining in practice. Being trusted refers to the school children considering the nurses to be reliable individuals who would listen to their innermost thoughts that they had not told anyone else. Listening to what the children had to say and their stories could be quite hard, but still gave the nurses pleasure, as it confirmed their trustworthiness as someone to be confided in. One nurse said:

School children may come to my office and test whether it’s safe to talk with me and if they can trust me. Some trust me at once, and for others it may take more time. It makes me really happy when I see that I have obtained their trust. Sometimes I can lose their trust, and without being trusted there’s nothing I can do. (School nurse 5)

Being trusted increased the nurses’ chances of being in a position to help the school children solve their problems. The nurses gave a lot of themselves in establishing a trustful relationship and in taking care not to lose this trust. In return, the nurses attained pleasure for themselves, which was what the nurses appreciated. For one nurse, this was expressed as experiencing a deep feeling – what she called ‘a resonance’ in return for her nursing practice:

I like being a school nurse, and I acknowledge that if there was nothing in it for me, I might have looked for another job. I haven’t thought about it in this way, but I give a lot of myself, and if I don’t receive something back, like a resonance, I don’t think I would remain. I believe it’s much more important than I previously thought. (School nurse 11)

Although this nurse did not often reflect on or talk about what she received for herself, she explicated that there must be something embedded in school nursing for herself. Without being trusted and attaining pleasure for herself, she might leave the practice.

#### Being given a response

For the school nurses, being given a response was another source of attaining pleasure for themselves. The nurses were given both verbal and non-verbal responses from school children. A verbal response could be an expression like ‘thank you’. Sometimes they were also given flowers or chocolate. Mostly, they received non-verbal responses in the form of the children’s spontaneous reactions, such as body language and gestures. This could involve receiving a hug or a smile or achieving eye contact with children who were reserved and shy. Such responses, confirmed the nurses had received a response related to what they had set out to do, for example, in creating a safe and calm atmosphere for the school children to relax in. One nurse described receiving a response as magical. She said:

It makes me happy to see when school children manage to relax or maybe they lay down on the sofa, which indicates they’ve calmed down and feel safe. It also feels like magic when the ones who hide their faces in their hoodies raise their eyes or smile at something I say or question. Such a response from the children gives me great pleasure. (School nurse 3)

Another nurse explicitly related being given a response to a feeling of success. Such a feeling arose when she experienced mastery in making a positive difference to the school children, which in turn encouraged her to remain in practice. She said:

It feels good when school children are grateful for seeing me. It motivates me when I know I have managed to make a difference for them. Thinking “YES – I managed” means a lot to me and is like a driver to hold on and remain, this makes me look forward to going to work. (School nurse 14)

Occasionally, the nurses received a negative response from the school children, in that they could become upset and refuse to talk with them. A similar thing could happen with the children’s parents, who could become angry with the nurses if, for example, they contacted the municipality child welfare service or the police about child abuse or poor parenthood. One nurse described a situation with parents who were angry with her and gave her a negative response, even though she had the child’s best interests at heart. She said:

There were some parents who were angry with me and felt that I had offended them. That was enormously hard for me. I knew I hadn’t done anything wrong, and I told them: ‘All I want is what is good for your child’. But they didn’t believe me. Then I started to cry. I found it very difficult that they didn’t believe my intentions were good. (School nurse 9)

Receiving negative responses was hard for the nurses, but more often than not, the responses of both school children and parents were positive and represented a source of pleasure for them.

The nurses also received positive responses from their nursing colleagues and school staff. The nurses were often complimented for sharing their knowledge and health perspectives on the situation in which school children find themselves. In this way, the nurses were praised for their professional initiative and contributions. The nurse in the above example emphasized this and said:

The school staff give me a lot of positive responses like: ‘It was so important that you took part in this meeting.’ ‘What you said, your focus, was really important’. It’s a good feeling knowing that I’m needed among the school staff too. If I’m to remain in practice, it’s important to have a team of good and supportive people around me. (School nurse 9)

Receiving a positive response from colleagues was a ‘good feeling’ for this nurse and she attained pleasure related to being supported by colleagues, which again was seen as related to remaining in practice.

## Comprehensive understanding and discussion

This study’s results demonstrate that what it is the school nurses receive for themselves that influences their remaining in practice is ‘gaining interesting workdays’ and ‘attaining pleasure’ for themselves. They gain interesting workdays with an ‘attractive scope of practice’ and ‘varied tasks’, as well as workdays where they attain pleasure by ‘being trusted’ and ‘being given a response’. This means the school nurses have workdays they enjoy. The themes of this study can be comprehensively understood as an expression of what the school nurses identify as the main locus of the good work-life. The school nurses’ remaining seems to revolve around what it is they receive on their own behalf: an affirmation for their ordinary life and what they do as a nurse. This understanding can be further elucidated in the light of the Canadian philosopher Charles Taylor’s [42] notion of ‘affirmation of ordinary life’, that implies identifying “the main locus of the good life” (42, p. 23). Taylor states that in modern culture, the main locus of the good life can be found in “the life of production and reproduction, of work and the family” (42, p. 23). Identifying the main locus of the good work-life requires what Taylor calls “insight about the value of ordinary life” (42, p. 24). Such insight regards what is inherently good in life, meaning the main locus of the good life has to do with an emotional satisfaction as “our emotions make it possible for us to have a sense of what the good life is for a subject” ([43], p. 65). In turn, being oriented towards what is inherently good in life contributes to an affirmation of ordinary life or what Taylor describes as “what would be a rich, meaningful life” (42, p. 42).

Accordingly, this study adds to previous research by demonstrating that remaining in practice revolves around what the nurses receive on their own behalf. Comprehensively understood as their being able to identify the main locus of the good work-life and orienting towards what is inherently good in life, this is what contributes to their receiving affirmation of their ordinary work-life and what they do as nurses. This is a finding in line with previous research indicating that what school nurses receive on their own behalf is interesting workdays, which they appreciate, as they can make a difference to school children’s lives [31, 32, 44]. School nurses are grateful for the opportunity to be trusted by school children and find this essential in order for them to succeed in their nursing practice [27, 31, 44, 45]. This outweighs what can be difficult and negative in their work-life [32, 33]. Previous researchers have stated that nurses in other fields of practice perceive their work-life as meaningful for themselves, as it involves making a difference for patients [15, 17]. Such a work-life gives zest and energy, as the nurses receive kindness and gratitude [10, 14]. Nurses say that “they get more back from patients than they give to patients” as they “receive self-gratification through giving” (10, p. 727). This indicates that nurses identify their work-life to be good in creating what is good, both for the patients and for themselves [9, 19]. Thus, looking after and providing what is good for the patients is good for the nurses’ own satisfaction [11]. Experiencing such satisfaction can be related to identifying the main locus of the good work-life – in other words, the school nurses’ understanding of “what it is that makes this job so good for me” (34, p. 660). Understanding this is linked to nurses’ insight into what it means to be a nurse and what the profession entails [10, 46].

Even though research demonstrates that school nurses may feel ‘stretched’ by the diversity of tasks [29, 30, 47], it is reported that they find workdays with opportunities for more learning to be rewarding [31, 33]. Collaborating in interdisciplinary teams and combining efforts with school staff strengthens the school nurses’ skills and contributes to their learning from other professions [29, 47]. Through interdisciplinary collaboration, the school nurses receive support and response for what they do [47, 48]. Receiving such a response is reported to generate nurses’ feeling of success and engagement in their workdays [13, 14], which influences satisfaction and remaining in practice [49] and strengthens nurses’ professional pride [12]. However, school nurses often have to advocate for inclusion in interdisciplinary teams, as other professionals may not be aware of their role and what they can contribute [29, 47].

Stating that what nurses receive on their own behalf is experienced as emotional satisfaction, can be associated with what Sanders [16] describes as a sense of ‘muchness’. Muchness is related to nurses’ experience of “well-being”, which arises when the nurses provide nursing practice “in accordance with an individual’s values and beliefs” (16, p. 2). The current study is thus in line with previous research, which demonstrates that nurses remain in practice due to an *affective* response to work [50] and also when they fulfil what they identify to be of worth for themselves [19] and take a stand for what is good for themselves [34]. What previous research indicates here in regard to remaining in practice can be seen as related to what the current study describes as the school nurses identify as the main locus of a good work-life for themselves. Lisk [51] has suggested that school nurses’ affirmation of their work-life concerns accentuating the positive in nursing practice and is important for their remaining in practice. This supports the current study in demonstrating that the school nurses’ affirmation of ordinary life as a nurse influences their remaining in practice.

### Strengths and limitations

Although we acknowledge that school nurses’ remaining in practice is influenced by other aspects than what they receive for themselves, this study provides a unique perspective, as the participants represented a specific group of nurses who tend to remain in practice. The strength of the study relates to highlighting that nurses receive something in return for themselves and it is specifically this *something* that may influence their remaining in practice. When considering the relevance of this study, it is important to note that the field of school nursing differs somewhat from other nursing practice, which might have generated other findings. Another demographic data set with younger or male nurses, or nurses of other nationalities, could also have generated other findings, as younger nurses tend to have a higher degree of turnover [50] and male nurses might have other expectations about what they receive for themselves. Due to different intervals (2–9 weeks) between the two interviews, some of the participants had less time for their reflections about the study aim to mature. The data collection nevertheless resulted in what we consider to be a rich and nuanced data material.

## Conclusion

This study describes and interprets what it is school nurses receive for themselves and that influences their remaining in practice. It highlights that the school nurses gained interesting workdays and attained pleasure for themselves, which can be comprehensively understood as an expression of what they identified as the main locus of a good work-life for themselves. By identifying the main locus of a good work-life, the school nurses received affirmation for their ordinary life and what they do as a nurse.

The study has relevance for nurses working in settings other than school health, as the results demonstrate that nurses receive something on their own behalf that may influence their remaining in practice. It is therefore important that nurses identify the main locus of their good work-life to receive affirmation for what they do in their ordinary work-life. Such affirmation may influence remaining in practice. However, more research is needed to study what nurses in other settings or demographics receive for themselves and how this may influence their remaining in practice.

## Data Availability

The datasets analysed during the current study are not publicly available for reasons of sensitivity but are available from the corresponding author on reasonable request.
